# Joint perceptual decision-making: a case study in explanatory pluralism

**DOI:** 10.3389/fpsyg.2014.00330

**Published:** 2014-04-23

**Authors:** Drew H. Abney, Rick Dale, Jeff Yoshimi, Chris T. Kello, Kristian Tylén, Riccardo Fusaroli

**Affiliations:** ^1^Cognitive and Information Sciences, School of Social Sciences, Humanities and Arts, University of CaliforniaMerced, CA, USA; ^2^Center for Semiotics, Aarhus UniversityAarhus, Denmark; ^3^Interacting Minds Center, Aarhus UniversityAarhus, Denmark

**Keywords:** explanatory pluralism, philosophy of science, joint decision-making, alignment, complexity matching

## Abstract

Traditionally different approaches to the study of cognition have been viewed as competing explanatory frameworks. An alternative view, explanatory pluralism, regards different approaches to the study of cognition as complementary ways of studying the same phenomenon, at specific temporal and spatial scales, using appropriate methodological tools. Explanatory pluralism has been often described abstractly, but has rarely been applied to concrete cases. We present a case study of explanatory pluralism. We discuss three separate ways of studying the same phenomenon: a perceptual decision-making task (Bahrami et al., 2010), where pairs of subjects share information to jointly individuate an oddball stimulus among a set of distractors. Each approach analyzed the same corpus but targeted different units of analysis at different levels of description: decision-making at the behavioral level, confidence sharing at the linguistic level, and acoustic energy at the physical level. We discuss the utility of explanatory pluralism for describing this complex, multiscale phenomenon, show ways in which this case study sheds new light on the concept of pluralism, and highlight good practices to critically assess and complement approaches.

## INTRODUCTION

Behavioral and cognitive processes are complex phenomena spanning multiple scales of organization, which may require multiple approaches to be fully understood. However, researchers have often aimed for a singular, unifying paradigm in the study of cognition (e.g., Fodor, 1975; Port and Van Gelder, 1995). The “paradigm wars” in cognitive science originated in the notion that one, or perhaps a limited number, of theoretical accounts will turn out to be most appropriate for the study of cognition. Herein, we will argue that multiple approaches should be used to study cognition at different scales of analysis. We consider a specific case study in detail, and show how, in practice, distinct methodological tools can be used to understand the same phenomenon in greater detail than any single paradigm could alone.

We begin this article by reviewing the history of reductionism and anti-reductionism. We then describe a third intermediate view, explanatory pluralism, which advocates the complementary use of more than one perspective at once, and has emerged as a way of studying complex systems in physics, biology, and other areas (Dale et al., 2012). This view, we argue, is especially well suited to the study of multiscale behavioral and cognitive phenomena (Ihlen and Vereijken, 2010; Kello et al., 2010; Dixon et al., 2012). We identify two benefits from practicing explanatory pluralism – *top-down constraining* and *bottom-up scaffolding* – and illustrate them through a case study of explanatory pluralism. We describe three empirical investigations of the same phenomenon at different levels of analysis, and from different theoretical perspectives. We end by considering how to critically assess and complement approaches, what is gained in this case by the pluralist approach, and what would be lost by more traditional reductive and non-reductive approaches.

## MULTISCALE NATURE OF COGNITIVE AND BEHAVIORAL PHENOMENA

A clear example of the need for a plurality of approaches is to be found in the multiscale nature of cognitive and behavioral processes. Visual recognition happens through rapid millisecond dynamics of neural population codes in the brain (Mauk and Buonomano, 2004). However, precisely the way this happens is shaped on the longer timescales of ontogenesis and cultural evolution. For instance, sensitivity to certain color distinctions seems largely influenced by linguistic inheritance (Roberson et al., 2005; Winawer et al., 2007) and even the famous Müller-Lyer illusion has been found to be modulated by the saliency of carpentered corners in a given culture and environment: infants growing up in some cultures will be more prone to perceive all angles as square corners distorted by distance (Henrich and McElreath, 2003; Henrich et al., 2010). It is increasingly acknowledged that cognitive and behavioral phenomena generally involve multiple temporal and spatial scales (e.g., Newell, 1994; Dale et al., 2012).

As a working definition, we can define the scale of a method as the set of units typically used in analyses. On this definition the temporal scale of neural activity tends to emphasize a milliseconds-to-seconds range, while the temporal scale of geology tends to emphasize a kiloannums-to-gigannums (thousands to billions of years) range (cf. Newell’s “Bands of Cognition”: Newell, 1994). The spatial scale of a discipline or method relative to a phenomenon can be defined in a similar way. Neuroscience works mainly in the nanometer-to-centimeter scale, while ecology considers environments on a meter-to-kilometer scale. It has to be noted that a discipline or method can consider multiple spatial and or temporal scales, as well as relations between them: ecologists, for example, sometimes consider the relationship between relatively low-level chemical processes in the soil of a region and higher-level processes like the viability of species in that region; and of course, physics considers everything from the smallest scales of particle physics to the largest scales of the cosmos as a whole.

A prime example of a complex, multiscale cognitive and behavioral phenomenon is human language (Beckner et al., 2009). Units of language such as phonemes, syllables, words, phrases, texts, and discourse exist at distinct scales. They are studied at corresponding temporal and spatial scales, from raw acoustic energy patterns unfolding in the milliseconds range, to larger structures encompassing minutes, hours, and even days. The range extends further still, to the slower pace of language change and evolution that occurs over years and centuries. These different scales are studied using a variety of different frameworks and methods, including Fourier analysis, Markov chain analyses, discrete- and continuous-unit power law analyses, and for language in particular, corpus methods and semantic analyses. Linguistic behavior has been shown to be systematically organized across multiple time scales. Phonological distributions, word frequencies in a given language, and sequences of words in texts all follow power law distributions, where the frequencies of a given unit are in proportion across multiple scales of analysis (e.g., Zipf, 1949; Ferrer i Cancho et al., 2004; Kello and Beltz, 2009; Altmann et al., 2012).

As a consequence, we argue that no cognitive or behavioral phenomenon can be exhaustively described by reference to a single temporal or spatial scale or theoretical framework. The question thus should not be *which one scale* of analysis or *which one theoretical framework* is the right one to target and study for a given phenomenon. Rather, the issue is *which scales* and *which theoretical frameworks* are relevant for the question at hand, and *how they relate to each other*. This is the essence of the position called “explanatory pluralism.” The alternative and more traditional account, which we delineate in more detail below, would be to focus on one scale of analysis and/or one theoretical framework for each given scale of phenomena.

## EXPLANATORY PLURALISM

Contemporary philosophy of science grew out of the logical positivist movement of the 1920s, according to which the only meaningful statements are those that can be empirically verified. Psychology, for example, was taken to be meaningful only insofar as its statements could be translated in to the verifiable statements of a physical language (Carnap, 1959), and in that sense reduced to physics. This kind of view ran into various problems (e.g., the principle of verification seems to be meaningless according to its own criteria)^1^, but the overarching project persisted: to understand in a formally rigorous way what science is and how different sciences are related to each other.

A standard view among the logical positivists, which remained even after positivism went out of fashion, was reductionism (Oppenheim and Putnam, 1958; Nagel, 1961; Cat, 2013 for review). According to the standard “layer cake” version of reductionism, higher-level “special sciences” (e.g., chemistry, biology) are arranged into a hierarchy, from physics to sociology, with physics at the bottom. It was thought that all the statements of any sciences besides physics could be reduced to the statements of the next lower level science via a system of “bridge laws.” For example, one might assume that sociology would reduce to psychology, psychology would reduce to biology, biology would reduce to chemistry, and chemistry would reduce to physics. In this way, all empirical claims could ultimately be reduced to the laws of physics. This was the “reductionist” consensus until about 40 years ago^2^. It was also known as the “unity of science” view^3^.

The more radical proponents of reductionism were also “eliminativists,” who thought that, in light of ongoing reductions, all special sciences (psychology, economics, etc.) would be eliminated. In the end we would only need physics, because the other sciences are really just describing physical stuff using labels and other descriptive conveniences.

In the early 1970s the reductionist consensus came under attack. Fodor (1974), in an influential paper subtitled “the disunity of science,” challenged reductionism by arguing that, even if it is true that nature is in some sense organized hierarchically, with fundamental particles aggregating into larger and larger systems of particles, this does not entail that higher level “special sciences” will be eliminated or reduced. The special sciences are, for Fodor, *not* eliminable; they are “autonomous.” The reason is that higher-level theoretical vocabularies do not line up in tidy one-to-one ways with lower level theoretical vocabularies, the way the bridge-law approach suggested. A term like “desire” does not correspond one-to-one to a “natural kind” of neuroscience, since it is multiply realized in different kinds of organisms. Thus “desire” is a proprietary term of a distinctive science, psychology, which cannot be eliminated. Fodor’s argument and other similar arguments (e.g., Davidson, 1969; see Cat, 2013 for review) were highly influential, and “non-reductive physicalism” became the new consensus for at least a decade.

A key feature of non-reductive-physicalism was the idea that, in practice, autonomous special sciences need not interact with lower-level sciences. Economists don’t need to understand quantum field theory in order to study labor markets, even though labor markets are physical systems that obey the laws of fundamental physics. In fact, it would be a mistake, a waste of time, for an economist to consider such low-level phenomena. In a similar way, Fodor claims, psychology should describe laws of behavior and cognition without wasting time on the low-level “implementation details” of neuroscience^4^. So non-reductive physicalism is associated with a kind of theoretical segregationism or “siloism” (our term), whereby different sciences are different levels, which maintain a principled isolation from one another.

So we have two views: (1) reductionism, where all special sciences reduce to physics, so that (in extreme eliminativist forms of this view) all sciences can in principle be eliminated except physics, and (2) anti-reductionism, where special sciences remain autonomous, and (in extreme “siloist” forms of this view) need not consult one another to do their work.

Explanatory pluralism is an intermediate third view, where special sciences are taken to be *semi*-autonomous (Edelman, 2008; Dale et al., 2009; de Jong, 2010; Hotton and Yoshimi, 2010; Yoshimi, 2012)^5^. On this view, different sciences have a degree of autonomy (they are not to be eliminated), but also interact in an effort to understand physical reality at different scales (they are not fully autonomous silos). According to the form of pluralism we advocate, different sciences and theoretical approaches should maintain their emphasis on different proprietary scales but should *also* work to unify their work as much as possible, insofar as they often describe the same phenomena in different but compatible ways.

Consider the old story about the blind men and an elephant (Saxe, 1884). Each of a group of blind men feels a different part of an elephant and then comes up with an incomplete, incompatible account of it.

Six blind men encounter an elephant. Each feels a different part, and infers from the properties of the portion encountered the nature of the whole (one feels the tusk and concludes that he has encountered a spear, another feels the trunk and deduces that he has met a snake, etc.). It is often suggested that we are in the same position with respect to consciousness: different (even incompatible) theories may be derived from correct, but incomplete, views of reality. (Sloman and Chrisley, 2003, p. 4)

But with a little collaboration they can recognize that they are describing the same thing in different ways, and thereby collectively contribute to a fuller understanding of their target phenomenon.

Within cognitive science, variants of this pluralistic theme have a history, even if not by name. The concept of distinct “levels of analysis” goes back at least to Marr (1982), with his famous explanatory hierarchy of the computational, algorithmic, and implementational levels; each level with its own focused program of investigation. However, Marr’s (1982) work was appropriated by Fodor and others to support strong forms of autonomy, which discourage interaction between theories at different levels. Just as software engineers don’t need to understand the implementation details of the computer that runs the algorithms they write, so too psychologists don’t need to understand the neural hardware that implements the algorithms and computations they describe^6^.

Approaches advocating more pluralistic interactions between theories emerged in the 1980s and early 1990s, as researchers began to develop ways of unifying connectionist and symbolic approaches to cognition in common frameworks (e.g., Smolensky, 1988; Bechtel, 1990). More recently, a variety of theorists have developed frameworks for integrating different approaches to cognition. One example is the area of symbolic dynamics, where the lower-level dynamics of a system can be “coarse-grained” (multiple states at a lower level are treated as a single state at a higher level) and thereby analyzed in terms of discrete computational states (Dale and Spivey, 2005; Edelman, 2008; Yoshimi, 2010). These types of approaches allow researchers to study systems using multiple theoretical frameworks (e.g., dynamical systems theory and finite automata theory), but also to study the relationship between these theories (e.g., Crutchfield, 1994; Shalizi and Crutchfield, 2001; beim Graben and Potthast, 2009; Atmanspacher, 2011; Butterfield, 2011; Yoshimi, 2012; Dale and Vinson, 2013^7^.

Explanatory pluralism does not imply the anarchistic idea that “anything goes”^8^: often, more than one approach is needed, but not all approaches are equally motivated, and many are even not warranted. If two approaches contribute the same (or largely correlated) information about a phenomenon, they should be treated as competing alternatives. In such a case, either one will produce a better explanation (and the other is a mere symptom, which can be discarded), or it might turn out that they are both driven by a third factor that needs to be identified. A critical criterion for explanatory pluralism is thus that the multiple approaches should not only be motivated by complementary perspectives, but should also contribute different and independent (minimally correlating) information about the subject matter. The cumulative addition of approaches to a research question is only justified to the extent that each new approach enables the researcher to account for new aspects of the phenomenon that would be inaccessible given other approaches. Comparisons between approaches are also necessary in order to assess their reciprocal productivity and explanatory power. This can be done in at least three often related ways: (1) through a conceptual analysis of the approaches involved, (2) through a data-driven statistical model comparison, and (3) through a more direct experimental manipulation of the factors involved, aimed at disentangling the reciprocal role of the mechanisms suggested by the different models.

The current case study is an example of a conceptual analysis of explanatory pluralism. In this case there is no explicit model fitting or experimentation across levels, but rather a theoretical analysis of how multiple independently motivated analyses of the target phenomenon, framed at different temporal and spatial scales, are related to one another. If the role of the scientist is to investigate, observe, and continually add to explanations of phenomena, it seems obviously valuable to show how multiple observations and theories, despite differences in method and scale, can be complementary. A conceptual analysis can take different forms depending on the specific features (e.g., types of analysis) of the theories being integrated. The main idea is that there is a synthesis of results from various levels of analysis. The way to go about synthesizing depends in part on the type of analytic practice involved in the theories being synthesized, which we discuss later in this section.

A more direct way of applying explanatory pluralism is by using data-driven analysis. This requires the utilization of model-fitting procedures (e.g., stepwise linear regression indices such as adjusted R-squared, log-likelihood, AIC, BIC; see Schwarz, 1978; Hastie et al., 2009; see also Myung and Pitt, 1997) and, most importantly, commensurate units of data from multiple levels of analysis. Under this strategy, the question becomes: how much variance of the phenomenon does each level of analysis explain? Although this strategy might seem to be most optimal for “compatible” (Giere, 2004) levels of analysis, issues of measurement error and methodological assumptions can become limiting factors that need to be addressed. Related to data-driven practice is the experimental practice of carefully manipulating parameters in order to discriminate between “causal” roles of different mechanisms^9^.

The third approach – designing experiments to test competing theories – is quite common in the realm of cognitive psychology, in which behavioral data can be leveraged against theoretical sticking points. By directly testing the predictions of potentially competing theories, an experimenter might confirm one theory or disconfirm another. This strategy is perhaps the most common approach to the theoretical sticking points in cognitive science. Famous recent examples include the “past-tense debate” (Pinker and Ullman, 2002) in psycholinguistics, or “prototypes vs. exemplars” in categorization research (Rouder and Ratcliff, 2006), in which dozens if not hundreds of empirical papers have explored these topics. In general, however, the degrees of freedom available to a theory, and to an experimenter, make it very difficult to develop “critical tests” and the weight of evidence on one side or the other has to gradually accumulate. Incidentally, neither of the vigorously pursued debates cited in this paragraph has been resolved to consensus, but integrative approaches have indeed been proposed for some (e.g., Love et al., 2004).

Explanatory pluralism affords the scientist a method for developing *fuller* explanations of relevant phenomena. The question then becomes how to apply explanatory pluralism in practice. In practice, what techniques are available for analyses and explanations of a phenomenon that exists at multiple temporal and spatial scales? Though there is no universally agreed upon model of explanation (Woodward, 2009), we can make a start by describing several specific approaches to explanatory pluralism: *top-down constraining* and *bottom-up scaffolding*.

Top-down constraining affords the scientist a basis for unifying multiple levels of analysis by identifying longer-scaled levels as *contextual constraints* for the smaller-scaled levels. For example, the amount of phonetic convergence (Pardo, 2006) – the phenomenon where the phonetic properties of interlocutors tend to align over the course of an interaction – depends on the contextual properties such as participant role and sex of the dyad. The contextual properties, such as the role of a participant in a conversational task, constrain behaviors occurring at shorter timescales such as the phonetic repertoire of interlocutors. We are not asserting a problematic “downward causality,” but rather are describing a pattern of scientific practice. We are advocating that scientists identify and analyze the different types and levels of contextual influences on a phenomenon.

Bottom-up scaffolding provides a framework for identifying what can emerge from lower-level patterns (i.e., patterns existing at shorter time scales or smaller spatial scales), and the dynamics and processes by which these patterns are formed. It is the substrates of lower levels that allow higher-level phenomena to emerge. As with top-down constraining, we need not assert any kind of problematic cross-level causality. Bottom-up scaffolding provides the scientists with a means of expressing how (for example) symmetries at lower levels must be broken for distinct phenomena at higher levels to occur (Kugler and Shaw, 1990).

These ideas are inspired by the heuristic identity theory (HIT) proposed by McCauley and Bechtel (2001). In this theory, processes of bridging across levels of explanation are not a matter of simplistic isomorphism between laws, or mappings between ontologies. Instead, mapping across levels should create mutual constraint, in that levels should be consistent, if qualitatively, with each other. Mapping should also generate new questions, as each level may inspire new lines of investigation in the other. These two benefits of heuristic mapping may guide an eventual synergy between levels of analysis. “They enable scientists working at one analytical level to exploit the conceptual, theoretical, and methodological and evidential resources available at another.” (p. 743). HIT embraces both streams of influence proposed here: from top-down constraints and from bottom-up scaffolding.

Despite all this theoretical work supporting explanatory pluralism, there have been few if any detailed studies of specific cases. We fill this gap by considering a specific case in detail. In the case we consider, multiple frameworks are used to analyze the same data: a corpus of conversing individuals solving a joint decision-making task (Bahrami et al., 2010). We discuss three approaches: a systemic approach at the timescale of ~60–90 min in which the entire sequence of joint decisions is analyzed for its statistical properties, a lexical approach emphasizing the words spoken in the conversation at the timescale of minutes, and a physical approach focusing on the multiple time-scales of micro and macro coordination as expressed by the timescale of acoustic energy of participants’ speech events. Each approach is born from very different theoretical assumptions, and focuses on a different scale using different theoretical and methodological tools. No single approach fully encompasses the phenomenon of joint decision-making. However, by taking all three approaches into account, we argue, joint decision-making is understood in a more articulated way than if it were studied at just one scale or using just one methodology. This is our notion of explanatory pluralism: the synergy of multiple theoretical frameworks targeting various scales of analysis in the investigation of a particular phenomenon^10^.

## CASE STUDY: JOINT DECISION-MAKING

Most of us must work in groups to complete complex tasks such as organizing conference symposia and collaborating on research projects; the production of this manuscript is one such example. In the past decade, a substantial research literature has emerged focusing on the cognitive, neurocognitive, behavioral, and physiological effects of working collectively in pairs or groups (for reviews see Fusaroli et al., in press; Pickering and Garrod, 2004; Shockley et al., 2009; Cooke et al., 2012). However, there is still much debate about whether individuals perform better than pairs or groups, and if so, how and under which conditions (e.g., Rajaram and Pereira-Pasarin, 2010).

Bahrami et al. (2010) recently developed a paradigm for studying collective perceptual decision-making that begins to address questions of joint perceptual performance. The paradigm was inspired by models of sensory integration that address how individuals integrate information from different sensory modalities (Ernst and Banks, 2002). Their goal was to test the question: Would two people be able to integrate their perceptual information, as individuals integrate information from different senses, in order to optimize their decisions? In other words, would two heads be better than one, and in particular, better than the best individual performance in a pair? They found that when two people were given the opportunity to communicate freely about their level of confidence on a trial-by-trial basis, two heads *became* better than one. However, this collaborative benefit was dependent on the interlocutors being equally good at solving the task on their own: differently performing interlocutors would not benefit from collaboration.

We argue that this joint decision-making paradigm provides a concrete case study for assessing explanatory pluralism. The three studies discussed are semi-autonomous in that they originate from disparate theoretical perspectives and focus on very different time scales, but at the same time complement each other, increasingly building an understanding of how and when interlocutors gain a collaborative benefit.

### APPROACH 1: BEHAVIORAL/DECISION-MAKING (Bahrami et al., 2010)

**FIGURE 1:**
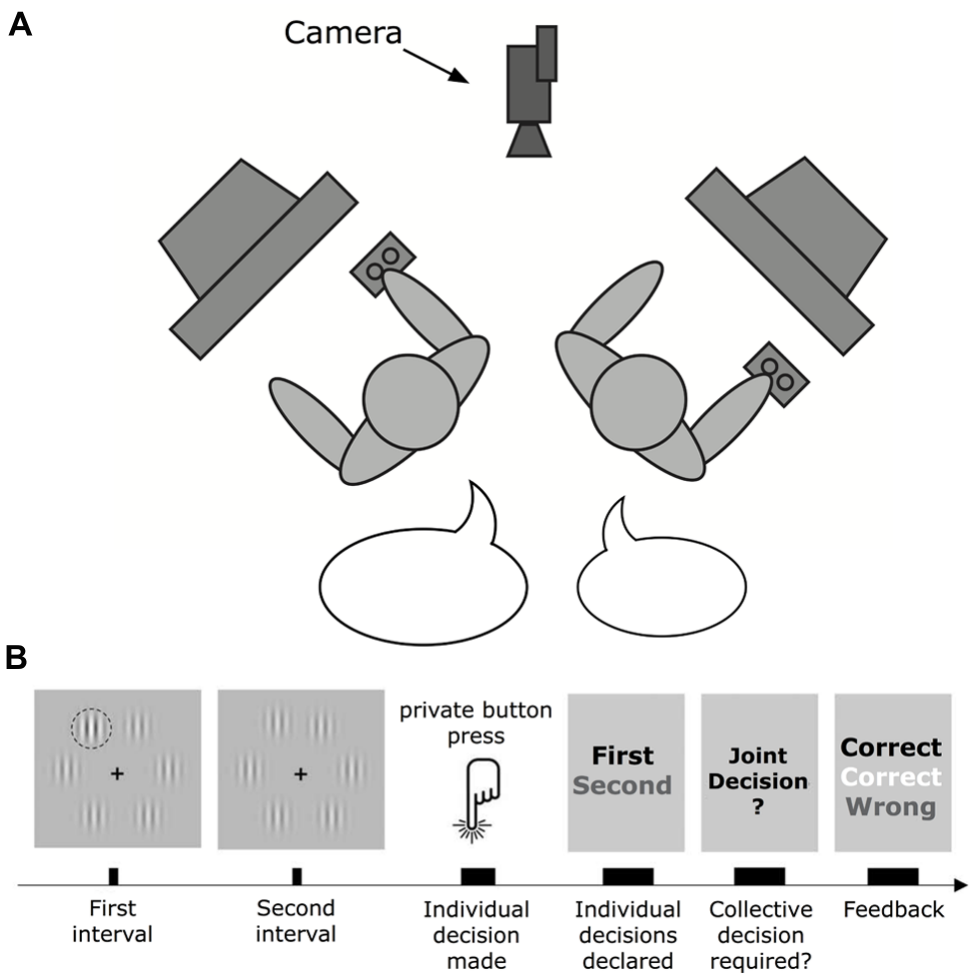
**Experimental setup (adopted with permission from Fusaroli et al., 2012). (A)** The experimental setup. **(B)** Schematic illustration of a typical trial.

In the original study (Bahrami et al., 2010), dyads were given a perceptual oddball task. The participants were recorded while sitting in front of their own respective screen at right angles to each other in a darkened room. The screens were identical and displayed exactly the same video output. On each trial the participants were sequentially shown two 85 ms long visual displays containing six Gabor patches. One of the displays would contain a contrast oddball: one of the six Gabor patches would have a stronger contrast and therefore look slightly darker (**Figure 1**).

The strength of the contrast varied randomly across trials. The participants were instructed to individually and separately indicate which of the displays contained this contrast oddball, by pressing a button. As long as both participants gave the same answer they would automatically proceed to the next individual trial. However, if their individual choices disagreed, they were prompted to negotiate, by freely discussing with each other, a joint decision. There was no time or other constraint on the joint decision dialogs. Individual and collective accuracy were then calculated by fitting a psychometric function to the dyad data^11^. The benefit of collaborating was then computed as the ratio between collective accuracy and the individual accuracy of the better of the two individuals.

Bahrami et al. (2010) used the empirical data thus produced to compare four models of information processing and transfer, each emphasizing different components of sensory processing, joint decisions, and communication: decision-making as relying on (1) a coin flip, (2) prioritizing the most perceptually competent group member’s decision, (3) the sharing of confidence on the individual decisions, and (4) the sharing of the full perceptual information on the stimulus. The best explanation for the empirical data was model 3 – the weighted sharing of confidence on the individual decisions. However, the collaborative benefit was dependent on similarity of individual sensitivities to the stimuli contrasts: in other words, differently performing interlocutors would not benefit from collaboration.

From our perspective, the approach employed by Bahrami et al. (2010) required the coarse-grained aggregation of behaviors from every trial: The overall unit of analysis was the psychometric function calculated on the full sequence of joint decisions per each individual and per each pair. Finding that “two heads were better than one” required the aggregation of local behavioral responses (decision-making) into the global perceptual sensitivity – operationalized as the estimated psychometric functions for each individual and each dyad.

Aggregating over the dynamics of a given decision process is common to theoretical approaches in cognitive science. Indeed, testing the predictions of some theories *requires* such aggregation of outcomes, rather than of processes. Consider, for example, Bayesian accounts of cognition (Chater and Oaksford, 2003, 2008; Chater et al., 2006). Bayesian approaches consider *distributions* over potential decisions or states; the only way this can be achieved is by aggregating a large number of decisions or behaviors and characterizing their distribution. By doing so, we are able to use the Bayesian framework to predict the longer-term properties of a decision process, and assess whether that process obeys certain principles of rationality or optimality. In this way, the Bayesian approach and associated behavioral methods target specific levels of analysis, e.g., the purposive/computational of Marr’s levels.

**FIGURE 2:**
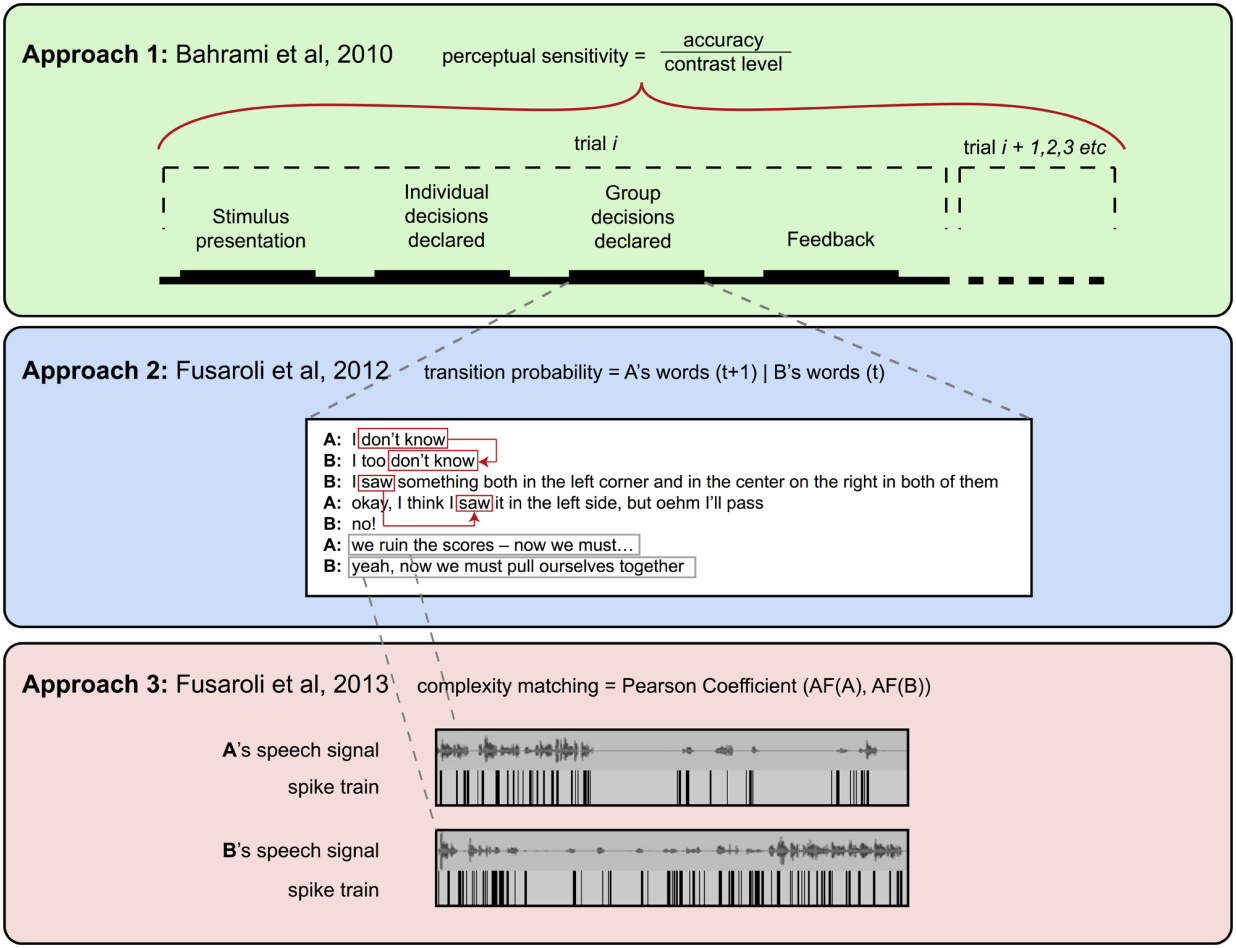
**The three approaches to decision-making paradigm discussed here, with an indication of their characteristic temporal and spatial scales as well as their favored methodological tools**.

The thesis of the original Bahrami et al. (2010) paper has these same properties. In order to assess the overall optimality of a joint decision process, we must aggregate over perceptual decisions. The underlying dynamics of the decision process (which we consider below) seem less relevant here; we want to know whether participants were interacting, and whether the presence of interaction (as a discrete variable) shaped their joint accuracy in interesting ways. Put simply, these questions require us to point to certain aspects of a task, aggregate over these decisions, and assess the outcome of our analyses with respect to predictions from these frameworks. See **Figure 2** for a diagram of the Bahrami et al. (2010) paradigm, which shows how different levels of the task are studied by the different approaches considered here.

### APPROACH 2: LINGUISTIC/CONFIDENCE (Fusaroli et al., 2012)

The second study we discuss had a very different starting point. During conversation, interlocutors have been observed to align to each other’s linguistic behaviors (Pickering and Garrod, 2004; Fusaroli and Tylén, 2012). The degree of linguistic alignment has been shown to have functional value; for example, high linguistic alignment tends to assist in some contexts of problem solving (Garrod and Anderson, 1987; Garrod and Doherty, 1994). However, it is disputable whether linguistic alignment is always beneficial and in what ways (for instance the extreme case of echolalia, where one interlocutor simply repeats what the other says, does not seem to be an effective conversational strategy; Fusaroli et al., 2014). Fusaroli et al. (2012) re-analyzed the Bahrami et al. (2010) experiment to explicitly investigate differences in conversational strategies employed by well- and poorly performing dyads. The aim was to map which aspects of linguistic alignment were functional for group performance in the joint decisions. This perspective required a finer-grained look at the actual process of decision-making, and not just on its results.

The videos of the joint decision-making tasks were transcribed. Following on Bahrami et al.’s (2010) insights that confidence sharing is crucial to effectively solve the task, the transcripts were coded for participants’ spontaneous ways of sharing confidence linguistically, for instance through expressions such as “I think I saw something.” Across the 16 dyads analyzed, 35 types of such confidence expressions were identified, e.g., modulations of “to see” as opposed to “to be sure.” Distributional patterns and token frequencies for each type of confidence expression were quantified in each transcript. *Local linguistic alignment* was calculated for all lexical items as the transitional probability of any given expression used by one participant being used by the other participant in the preceding joint decision. For example, local linguistic alignment was computed as the probability of Participant B using the expression “to see” when Participant A used the same expression in the previous trial.

By having indices of different types of linguistic alignment – one type that subsumes all lexical expressions (indiscriminate alignment) and one that subsumes only confidence expressions (discriminant alignment) – Fusaroli et al. (2012) were able to determine which type of alignment benefitted joint decision-making: task-specific, discriminate alignment or general, indiscriminate alignment. To measure the lasting effects of alignment, Fusaroli et al. (2012) also calculated a more coarse-grained measure of *global linguistic convergence* of confidence expressions. This was computed as the percentage of the overall sum of confidence tokens used by the dyad throughout the experiment, which belonged to the most frequent confidence type thus abstracting from the local linguistic exchanges between interlocutors and focusing on long term linguistic consistency of the pair.

Relying on Bahrami et al.’s (2010) psychometric function for calculating collective performance, Fusaroli et al. (2012) observed that task-specific, local linguistic alignment and global linguistic convergence positively correlated with collective benefit, whereas, local indiscriminate alignment negatively correlated with collected benefit. Furthermore, global convergence strongly predicted collective benefit: when dyads continually and consistently used shared sets of linguistic expressions for expressing confidence, collective benefit was observed to be higher. Fusaroli et al. (2012) concluded that in order for dyad members to benefit from cooperation they should not just parrot each other (indiscriminate alignment). Rather dyads that jointly adapted linguistic tools to meet the functional affordances of the task (sharing and comparing confidence) reached high collective performance.

Summing up, the search for functional linguistic alignment led Fusaroli et al. (2012) to conduct a corpus analysis of trial-to-trial transcripts highlighting the actual communication strategies employed to solve the joint decision task. Computing the transitional probabilities of lexical alignment required a fine-grained analysis of the local dynamics of lexical choices – the unit of analysis being lexical expressions within adjacent joint decisions – keeping track of individuals’ productions. It has to be noted that coarse-grained analyses were also crucial for the study: global linguistic convergence, collective benefit and even the aggregate measures of linguistic alignment to be used as dyad-level correlates for collective benefit. Importantly, the aggregation procedure applies at a different level of the analysis – at the finer-grained timescale of words being used by interlocutors.

As described above, aggregation in Bahrami et al. (2010) derived from a desire to quantify coarser-grained characteristics of the decision process: psychophysical sensitivity and the benefits of interaction. The Fusaroli et al. (2012) study provided insight into the mechanism and process of collective benefit, whereas the Bahrami et al. (2010) study provided a description of *why* these mechanisms work in a particular way. But, as discussed in background sections above, every theory has boundary conditions that limit the claims it can make about complex systems. Put simply, the aggregation approach is unable to specify both *how and why* the content or structure of an interaction helps joint decisions. Trying to get at these new aspects invokes more dynamic linguistic and psycholinguistic theoretical machinery, quickly leading to new questions and *different* levels of analysis. Instead of aggregating decisions alone, we “peel back” those decisions and peer into their contents, before aggregating in different ways from before.

### APPROACH 3: PHYSICAL/ACOUSTIC ENERGY (Fusaroli et al., 2013)

Recent work on “complexity matching” in the field of statistical physics has shown that information transmission between two complex systems is optimal when the complexities of the behaviors of the two systems match (West et al., 2008). Fusaroli et al. (2013) investigated whether such ideas would apply to human interactions (see also Abney et al., under review). Indeed, it has been shown that humans produce behaviors with long-range correlations at increasing time scales, and additionally, behaviors are observed to follow scaling laws evidenced by heavy-tailed distributions (Kello et al., 2010). Fusaroli et al. (2013) thus investigated if the statistical complexities of behaviors would match between two interacting humans, and if so, according to the physical models, the degree of complexity matching would predict information transfer to be optimal. Already Fusaroli et al. (2012) were looking for a functional relationship between the degree of matching of a particular behavior and the accompanying performance outcome; however, the use of complex systems physical models creates important divergences in the level of description and in the time scales of subsequent analyses.

To estimate the multiscale complexity matching of human behavior during interactions, Fusaroli et al. (2013) had to employ yet a new unit of analysis, capturing more basic perceptuomotor coupling between interlocutors. For this new unit they had to first assess the complexity (hierarchical scaling; cf. Abney et al., under review) and then the match in complexity between interlocutors, with the hypothesis that the more the complexity of participants’ speech behavior matched, the higher the collaborative benefit. They analyzed the physical basis or “skeleton” of linguistic exchanges: the acoustic energy of speech events of individuals in conversation. Onset/offset intervals of the acoustic signal from the conversations were extracted by identifying boundaries between speech and pauses (pauses were defined as reduced acoustic intensity and the absence of pitch lasting beyond 20 ms). Binary spike trains of speech events were computed from the onset/offset intervals; states were coded with “0s”; “1s” were used to code changes in state, that is, the onset or offset of a speech event. Thus, the unit of analysis was the onset/offset of a speech event defined by the presence or absence of particular properties of acoustic energy. A temporal estimate of complexity of human behavior was computed for each participant and each joint conversation trial employing Allan Factor (AF, Allan, 1966), a multiscale method for estimating the correlated clustering of speech events^12^. Complexity matching was defined as the degree of correlation between AF estimates of participants in a dyad. Across all trials, the authors found a positive correlation between degree of complexity matching and collective benefit: when the complexities of participants’ speech behaviors matched, collective benefit on the joint perceptual decision task was higher. These findings can be interpreted as preliminary evidence for complexity matching in interpersonal coordination (West et al., 2008): Increased collective benefit in a dyad can be considered an index of the optimality of information transfer, which increased as a function of the coordination of human behavior across multiple time scales. In other words, the more fine-tuned the turn-taking coordination of the interlocutors, the better the information transfer, which in turn led to a higher collective benefit. Crucially, the degree of complexity matching increased from the first to the second half of the experiment, suggesting that complexity matching express the degree to which interlocutors adapt to coordinate with each other.

This third study used a trial-by-trial measure that summarized the multiscale properties of speech coordination in its very basic form of acoustic energy. The overall unit of analysis was the onset/offset of acoustic energy at a 10 ms time scale. This fine-grained analysis was then aggregated into a coarse-grained analysis that operationalized the degree of complexity matching between dyads. The degree of complexity matching was then correlated with collective benefit computed for either all trials or session-by-session.

Again, the questions regarding the microstructure of coordination cannot come from linguistic analysis or from aggregation of perceptual decisions. Instead, it starts from a much more fine-grained level of analysis, specifically the dynamics of the perceptuomotor structure of the task. Just as theories about optimal decisions or linguistic alignment require selecting particular levels of description and analysis, here researchers choose a finer timescale and extract signals, which may be subject to their own (but different) aggregation. Researchers who adopt this theory often require very densely sampled behaviors to quantify and characterize the dynamics that underpin some behavior or cognitive process. Now quite different observations can be made about the process of interaction, regarding the composition of the task in terms of the perceptuomotor coupling of its participants.

The past three sections laid out sample re-analyses of the Bahrami et al. (2010) dataset, with quite different goals in mind. The upshot of this research scenario is to select disparate scales of analysis, different means of measurement, and different patterns of aggregation in order to assess particular predictions about the cognitive system, or to characterize the cognitive system in different ways at different levels. However, we do not mean to suggest that these are completely independent levels of analysis (*à la* Fodor, 1974, 1975; Fodor and Pylyshyn, 1988). Instead, these processes should be seen as interdependent, inspiring each other and providing reciprocal insight. Indeed, Fusaroli et al. (2012, 2013) already shows hints of this integration: The optimality of the joint decision process can be correlated with the structure of the linguistic interaction; similarly, the joint decision process and linguistic structure itself may be related to the dynamics of the acoustic speech behaviors of dyad members, such as the correlation between complexity matching indices. In the next section, we discuss this potential integration more fully, noting that explanatory pluralism also encourages this kind of synthesis across theoretical domains.

## SYNTHESIS OF LEVELS OF DESCRIPTION

Explanatory pluralism has been presented as a view intermediate between extreme forms of reductionism (where everything ends up being physics) and anti-reductionism or strong autonomy (where different sciences are insulated from one another). Above, we have seen a detailed example of explanatory pluralism in practice, with three different studies approaching the same phenomenon – joint decision-making by a pair of participants engaged in a perceptual discrimination task – at different temporal and spatial scales, using distinct methodological tools.

In this section, we return to the topic of explanatory pluralism, and consider how this kind of approach works in practice. First we discuss the benefits of each level of description. Then we discuss advantages of interactions between these levels, and how syntheses between them can motivate new questions and insights.

### BENEFITS OF INDEPENDENT LEVELS OF DESCRIPTION

Against views which emphasize the value of a single paradigm in cognitive science (e.g., reductionism in its most radical form, which advocates focusing only on the physical level), explanatory pluralism holds that it is important to study phenomena using multiple independent levels of analysis. We have seen how the three levels discussed above are important to understanding performance in joint decision-making. Bahrami et al. (2010) found that, when perceptual sensitivities were equal, dyads benefitted from interaction by comparing levels of confidence. Fusaroli et al. (2012) observed that group performance was higher when interlocutors shared common task-relevant lexical patterns during conversations about confidence. Finally, Fusaroli et al. (2013) provided evidence that group performance increased when the hierarchical structure of the very basic patterns of interlocutors’ vocalizations – with the base unit of the onset of acoustic energy – matched within the dyad. These insights were obtained while remaining within the relevant spatial and temporal scale and while making use of the characteristic tools of each approach.

What if a more traditional approach were followed, which only allowed for one way of analyzing joint decision-making? What would be lost?

If “Approach 1” were pursued in isolation, we would not know that the development and sharing of a linguistic confidence scale among members of a dyad makes a difference in the performance in the task (Fusaroli et al., 2012). Furthermore, the performance of the task is also successfully predicted by the hierarchical structure of acoustic energy onsets (Fusaroli et al., 2013).

If “Approach 2” were pursued in isolation, we would not know that the degree of matching in individual sensitivity is an important drive in the efficacy of confidence sharing. Additionally, we would not consider that the patterns of matching found in local linguistic alignment might be complemented by more basic patterns of matching of the hierarchical structures of acoustic energy onsets.

Finally, if “Approach 3” were pursued in isolation, we would not know that the rate of indiscriminate matching of lexical items negatively predicts performance; the alignment of language not functionally relevant for the particular context, does not help the dyad. Additionally, we would never be able to consider the possibility that unequal perceptual capabilities of individuals might have a significant effect on group tasks and relatedly, how, in turn, these asymmetries might affect linguistic-level and acoustic-level matching dynamics.

These considerations support the idea that strong forms of reductionism, which at times suggest outright elimination of all but the lowest level physical sciences, are problematic. All three approaches shed light on important features of joint decision-making. Eliminating any of these approaches leaves important features of the phenomenon unexplained. The synthesis of these three levels provides a more complete description of how people work together to solve a particular task.

### BENEFITS OF INTERDEPENDENT LEVELS OF DESCRIPTION

Against views like strong *anti-reductionism* or “siloism”, which advocate that different levels of analysis be *completely* autonomous, we believe that multiple theories should interact when describing the same phenomenon (cf. Simon, 1992). To make a case for this idea, we first consider what would be missing from a description of joint perceptual decision-making if the different approaches did not interact with each other.

If none of these three approaches informed each other, various research opportunities crossing scales and mixing methods would be lost. For example, can people with asymmetric perceptual capabilities effectively overcome their difference by communicating about common environmental constraints (Approach 1) and what do the language properties (Approach 2) look like when they successfully coordinate? Additionally, does local linguistic alignment or global convergence of linguistic coordination (Approach 2) relate to the matching of hierarchical structures at the level of acoustic energy onsets (Approach 3)? Finally, how does the degree of matching of hierarchical structures of acoustic energy onsets (Approach 3) relate to different dyad-level perceptual asymmetries (Approach 1)? All of these questions pertain to the corpus of data described in the present case study, and indeed some of these questions are currently being tested. What is important to understand is that all three levels are describing the same phenomenon, and there are certainly more levels of description that can be included. Asking these cross-level questions might persuade some to argue for reductionism, e.g., “does linguistic alignment just merely reduce to complexity matching?” or “how does linguistic alignment interact with dyad-level perceptual asymmetries?” We suggest that there is *interdependence* across levels, where theories can inform each other, ultimately leading to a better understanding of the phenomenon. Having an epistemological process prioritizing the interdependence across different levels stands in contrast to views suggesting that each level is independent, autonomous and represents competing explanations of a phenomenon and that higher levels can be reduced to lower levels.

### TOPICS FOR FURTHER WORK IN EXPLANATORY PLURALISM

We have argued for explanatory pluralism using a detailed case study. However, it is important to point out that not all levels of description are complementary, and the principle of “more levels of description is better” is problematic if applied haphazardly and without a proper supporting framework. While a plurality of approaches is necessary to better explain a given phenomenon, not all approaches are equal: At what point does the diffusion toward a troubled eclecticism stop? We have earlier suggested three methods: (1) conceptual analysis, (2) data-driven statistical model comparison, and (3) experimental manipulations. This paper is an example of the first: we have assessed three approaches to the same phenomenon (and dataset) articulating their differences and complementarity. A data-driven statistical model comparison – which is outside of the scope of this paper – would comparatively assess the relation between the models and between the models and the data. In other words, it would look at the comparative explanatory power of individual performance, linguistic alignment and complexity matching: Do they equally fit the data? Do they explain comparable amount of variance in the data? Do we get better statistical fit and explanatory power by integrating individual performance, linguistic alignment and complexity matching in the same model and how do they interact? An experimental approach could push these comparisons further by investigating the causal relations between parameters and opening new venues of inquiry. For example, if we systematically vary one of the parameters (e.g., similarity in individual performance, by introducing noise in the stimuli presented to the worse performer in a dyad), how do the others vary in their levels and relation to performance?

In conclusion, if the goal of a scientific endeavor (e.g., understanding decision-making of pairs of humans) is to fully understand and thus predict future behaviors, then taking into account multiple levels and theoretical approaches is warranted if not necessary.

We have provided a conceptual treatment of what explanatory pluralism looks like in cognitive science. Although beyond the scope of the current paper, we also advocate the use of data-driven statistical model comparison and experimental manipulations to critically assess the interest and complementarity of different approaches. Indeed, some of us (Fusaroli and Tylén, under review) have already implemented this technique.

Additionally, we introduced two benefits of* practicing* explanatory pluralism in scientific investigations: *top-down constraining* and *bottom-up scaffolding*. Both benefits provide frameworks that may lead to future research questions about a particular phenomenon. Again, top-down constraining unifies multiple levels of analysis by identifying the longer-scaled levels as contextual constraints for the smaller-scaled levels. For example, Approach 1 provided contextual constraints for the linguistic tools (Approach 2) participants might utilize, and therefore, the patterns of acoustic energy (Approach 3). This framework affords the identification of contextual influences of a phenomenon not otherwise integrated across multiple levels of analysis.

Bottom-up scaffolding can be used to identify what can emerge from lower-level patterns. For example, the lexical items participants jointly use and align (Approach 2) emerge from the multiscale patterns of acoustic energy (Approach 3). Furthermore, an optimal model of joint perceptual decision-making, requiring the sharing of confidence (Approach 1), is comprised of the lexical items (Approach 2). Again, it is the substrates of lower levels of analysis that afford the possibility for higher-level components – via higher levels of analysis – of a phenomenon to emerge.

## CONCLUSION

We have defended explanatory pluralism using a case study, which involved three separate analyses of the same phenomenon. We have made the case that integrating data and theory from multiple scales of analysis provides a fuller explanation of a cognitive phenomena than would be possible if we pursued a more traditional, theoretically autonomous style of scientific investigation.

Our call is for more researchers in the cognitive and behavioral sciences to consider studying phenomena of interest using the framework of explanatory pluralism. This can encompass a variety of practices ranging from conceptual analysis to full fledged, data-driven analysis. Acknowledging that theoretical approaches influence methodological decisions and practices, we argue that explanatory pluralism might be beneficial to the ultimate scientific endeavor of explanation.

## Conflict of Interest Statement

The authors declare that the research was conducted in the absence of any commercial or financial relationships that could be construed as a potential conflict of interest.

## Footnotes

1 The argument does not go through quite so easily, but there are still numerous problems with logical positivism and empiricism. For a detailed history and review, see Creath (2011) and Dienes (2008).

2 Reductionism (as well as anti-reductionism and other positions we consider) can be understood as ontological or epistemological theses. As an ontological thesis, reductionism, for example, corresponds to the physicalist claim that there is nothing over and above physical entities. Our emphasis here is on the epistemological theses, which concern (for example) whether and to what extent knowledge and understanding at higher levels requires knowledge and understanding at lower levels.

3 The concept of unity was actually understood in several, subtly different ways. See Cat (2013).

4 Fodor’s claim is that, assuming that a given pair of sciences (e.g., psychology and neuroscience) “cross-classify” the same phenomena, in the sense that they introduce predicates that do not map 1-to-1 onto each other (there is no isomorphism or set of “lawful coextensions” between them), then in practice it does not make sense for the two theories to interact (see Fodor, 1974, p. 113). Fodor clearly thinks it is a mistake to encourage cross-level interaction in the case of psychology and neuroscience: he bemoans the very idea of what would today be called “cognitive neuroscience”: “There are departments of ‘psycho-biology’ and ‘psychology and brain sciences’ in universities throughout the world whose very existence is an institutionalized gamble that lawful co-extensions can be found” (Fodor, 1974, p. 105). Such attempts are “foredoomed.” In another text Fodor and Pylyshyn argue at length against “brain-style modeling” in the cognitive sciences, and again treat it as a mistake, which they trace back to Lucretius: “the structure of ‘higher levels’ of a system are rarely isomorphic, or even similar, to the structure of ‘lower levels’ of a system. No one expects the theory of protons to look very much like the theory of rocks and rivers, even though, to be sure, it is protons and the like that rocks and rivers are ‘implemented in’. Lucretius got into trouble precisely by assuming that there must be a simple correspondence between the structure of macrolevel and microlevel theories…it seems that the commitment to ‘brain style’ modeling leads to many of the characteristic Connectionist claims about psychology, and that it does so via the implicit and unwarranted-assumption that there ought to be similarity of structure among the different levels of organization of a computational system.” (Fodor and Pylyshyn, 1988, p. 63). Though Fodor clearly abides by some kind of disciplinary isolation principle in such cases, subsequent non-reductive physicalists did not uniformly follow him in this (see, e.g., Sober, 1999).

5 There are differences between these views, but our focus here is on a generic form of explanatory pluralism which captures the general idea that no one science is proprietary and that multiple sciences are needed to understand physical reality in all its complexity.

6 We would not subscribe to this view of the relationship between software and hardware, at least in purist terms.

7 Within pluralism, we find that explanatory pluralism and truth pluralism have potentially interesting relationships. For example, Lynch (2001) provides arguments for a metaphysical pluralism countering questions of absolutism. Considering our proposal for an explanatory pluralism that sits between reductionist and anti-reductionist views, there is room for ample discussion on how epistemic and ontological pluralism can fit together. We hold our views on the matter for a future paper, as we consider this important topic to be out of the scope of the current paper.

8 Cf. Feyerabend (1975) “…there is only one principle that can be defended under *all* circumstances and in all stages of human development. It is the principle: anything goes.”

9 Both these latter practices could be profitably applied to the case in analysis, but escape the scope of the current paper.

10 Our review is inevitably selective. However, there are many variants of a pluralistic approach to science and cognition, in various domains, including: A rahamsen and Bechtel (2006), Atmanspacher and beim Graben (2009), Dennett (1991), Dupré (1993), Eliasmith (1996), Kellert et al. (2006), Kelso and Enstrøm (2006), Mitchell (2003), Weiskopf (2009), among others.

11 Psychometric functions were computed by plotting the proportion of trials in which the oddball match was reported in the second interval, as a function of the contrast difference with the surrounding patch array. The functions were fit with a cumulative Gaussian function. The slope of each function provided an estimate of perceptual sensitivity: the steeper the slope, the higher the sensitivity.

12 The Allan factor analysis computes the correlation estimate – α – of the variance of speech events at a particular time scale across multiple time scales. A scaling relation, or power law, of speech events is evidenced when α ~ 1 whereas, and α ~ 0 is considered a Poisson process. The complexity of a participant’s speech behavior is determined by the computation of α.
